# High Sulfation and a High Molecular Weight Are Important for Anti-hepcidin Activity of Heparin

**DOI:** 10.3389/fphar.2015.00316

**Published:** 2016-01-11

**Authors:** Michela Asperti, Annamaria Naggi, Emiliano Esposito, Paola Ruzzenenti, Margherita Di Somma, Magdalena Gryzik, Paolo Arosio, Maura Poli

**Affiliations:** ^1^Molecular Biology Laboratory, Department of Molecular and Translational Medicine, University of BresciaBrescia, Italy; ^2^G. Ronzoni Institute for Chemical and Biochemical ResearchMilan, Italy

**Keywords:** iron metabolism, hepcidin, low molecular weight heparins, 2-O and 6-O sulfated heparins, BMP6, anemia of chronic diseases

## Abstract

Heparins are efficient inhibitors of hepcidin expression even *in vivo*, where they induce an increase of systemic iron availability. Heparins seem to act by interfering with BMP6 signaling pathways that control the expression of liver hepcidin, causing the suppression of SMAD1/5/8 phosphorylation. The anti-hepcidin activity persists also when the heparin anticoagulant property is abolished or reduced by chemical reactions of oxidation/reduction (glycol-split, Gs-Heparins) or by high sulfation (SS-Heparins), but the structural characteristics needed to optimize this inhibitory activity have not been studied in detail. To this aim we analyzed three different heparins (Mucosal Heparin, the Glycol split RO-82, the partially desulfated glycol-split RO-68 and the oversulfated SSLMWH) and separated them in fractions of molecular weight in the range 4–16 kD. Since the distribution of the negative charges in heparins contributes to the activity, we produced 2-O- and 6-O-desulfated heparins. These derivatives were analyzed for the capacity to inhibit hepcidin expression in hepatic HepG2 cells and in mice. The two approaches produced consistent results and showed that the anti-hepcidin activity strongly decreases with molecular weight below 7 kD, with high *N*-acetylation and after 2-O and 6-O desulfation. The high sulfation and high molecular weight properties for efficient anti-hepcidin activity suggest that heparin is involved in multiple binding sites.

## Introduction

Iron is essential and potentially toxic, thus its homeostasis needs to be tightly regulated. In mammals the control of the systemic iron regulation relies mainly on hepcidin, which controls iron availability by suppressing ferroportin, the iron exporter (Ganz and Nemeth, 2012). Hepcidin expression, in turn, is regulated by systemic and hepatic iron stores, by erythropoietic activity, ER stress, hypoxia and inflammatory conditions (Hentze et al., 2010), with mechanisms that involve primarily the BMP/SMAD signaling pathway (Babitt et al., 2007). Among the various BMP members, BMP6 is the one dedicated to hepcidin expression, it binds to BMP receptors of type I (ALK2 and 3) and type II (AcvR2 and BMPR2) causing the phosphorylation of SMAD1/5/8 which assemble with SMAD4 and transfer into the nucleus to activate hepcidin promoter (Core et al., 2014). The mechanism includes other actors, like hemojuvelin (HJV), a GPI-anchor protein, that acts as BMP co-receptor (Babitt et al., 2006) and the serine protease TMPRSS6 that controls HJV activity. The inflammatory response induces hepcidin expression via the IL6-dependent activation of JAK/STAT3 signaling (Verga Falzacappa et al., 2007). Abnormal regulation of hepcidin expression occurs in various pathologies: when too low it leads to iron excess as in hemochromatosis (Zhao et al., 2013), when hepcidin is too high it leads to iron restriction, as in Iron Refractory Iron Deficiency Anemia (IRIDA) and in the Anemia of Chronic Disease (ACD) (Poli et al., 2014c). ACD is a common form of anemia associated with degenerative diseases including cancer, chronic kidney disease and rheumatoid arthritis. The pharmacological controls of hepcidin should improve the treatment of various disorders of iron metabolism, including ACD. Different approaches are in study for treatments to control hepcidin (Ganz and Nemeth, 2011; Fung and Nemeth, 2013; Poli et al., 2014c) and among them we found that heparins strongly repress liver hepcidin expression *in vitro* and *in vivo* (Poli et al., 2011, 2014a) by interfering with BMP6/SMAD1/5/8 phosphorylation and signaling (Poli et al., 2014a). The finding that this occurs also with heparins without anticoagulant activity strongly facilitated their use *in vivo*. For example, the glycol-split heparins (gs-Heparins) (Poli et al., 2014a) and the oversulfated heparins (SS-heparins) suppress hepcidin and increase iron availability in mice (Poli et al., 2014a,b) with no adverse effect. However, the heparin preparations normally used are heterogeneous and composed by molecules with different molecular weight and degree/topology of sulfation and their use do not provide precise indications on the chemical properties for optimal anti-hepcidin activity. To this aim we prepared and analyzed more homogenous heparin preparations with defined molecular weight and that have been treated to remove sulfate groups from oxygen 2 of iduronic acid (2-O) and from oxygen 6 of glucosamine residues (6-O). The results show that the anti-hepcidin activity is associated with molecular weight above 7 kD and with the 2-O and 6-O sulfation.

## Materials and Methods

### Heparin Derivatives

Heparin is an API porcine mucosal Heparin (PMH) (Bioiberica lot F415-10/0001). The chemically modified non-anticoagulant heparins RO-82 and RO-68 obtained by oxidation and reduction, and the 2-O and 6-O desulfated heparin samples were prepared as described in (Naggi et al., 2005). They differ in the degree of sulfation and the number of glycol-split residues for chain. Fractions with different molecular weight of samples MH, RO-82, RO-68, SSLMWH-19 were obtained by gel filtration through Ultrogel ACA54 (Sigma Aldrich) column (1.5 cm × 84 cm) equilibrated in 0.25 M NH_4_Cl. All samples were recovered after desalting on TSK HW 40S column (Tosoh Bioscience, Belgium) then were freeze-dried and characterized by NMR spectroscopy, (Guerrini et al., 2005) by conductimetric determination of SO_3_/COO- ratio (Casu and Gennaro, 1975), and by determination of average molecular weight (Bertini et al., 2005). Characteristics of the samples are reported in **Table 1**.

**Table 1:** Average molecular weight (Mw) rounded to 100s D of the fractions obtained from four different heparin types: the unmodified Mucosal Heparin (MH), the 30% glycol-split RO-82, the partially desulfated 42% glycol-split RO-68 and the ovesulfated SSLMWH-19.

	Mw (kD)			
Sample	MH	RO-82	RO-68	SSLMWH-19
Parent	17.20	16.00	16.40	8.80
Fraction 1	21.60	12.00	7.80	12.90
Fraction 2	14.40	9.20	6.20	10.30
Fraction 3	10.00	7.80	3.90	6.90
Fraction 4	–	6.80	–	4.00

### Cell Culture

The human hepatoma cell lines, HepG2 (from IZSLER, Brescia, Italy) were cultured in minimum essential medium (MEM, Gibco from Life technologies), 10% endotoxin-free fetal bovine serum (Sigma–Aldrich), 0.04 mg/mL gentamicin (Gibco), 2 mM L-glutamine (Gibco), and 1 mM sodium pyruvate (Carlo Erba) and maintained at 37°C in 5% CO_2_.

### Mice

The study was approved by the Institutional Animal Care and Use Committee of the University of Brescia, Italy. C57BL/J6 mice (Harlan Laboratories) were kept on a standard diet until 8 or 9 weeks. Four mice per experimental group were used for the treatment mentioned below, treated subcutaneously (SC) with saline or different heparins.

### Treatments with Heparins

#### In Vitro

Heparins were dissolved in sterile saline buffer at a concentration of 10 μg/μl for *in vitro* studies and stored at 4°C. Just before using, serial dilutions of them were prepared (33-11-3.6-1.2-0.4-0.12 μg/ml) in culture medium and they were added to the cells in the presence or the absence of BMP6 (10 ng/ml) (R&D) for 6 h. After the treatment the cells were collected and RNA extracted as reported below.

#### In Vivo

Heparins were dissolved in sterile saline buffer to the proper concentration to inject SC in the mice of the selected amount in a volume of 100–150 μl/mouse. The control group was treated with the same volume of sterile saline buffer. After 6 h the mice were euthanized, the liver RNA extracted and analyzed for mRNA of hepcidin, Id1 and Hprt1.

### RNA extraction and Quantitative qRT-PCR

After the treatments, total cell RNA was recovered with TRI Reagent (Sigma–Aldrich), according to the manufacturer’s instruction. Reverse transcription was performed using 1 μg RNA, oligo dT, and Improm Reverse Transcriptase (Promega) in 20 μL. Samples (1 μl) were used for quantitative reverse transcription polymerase chain reaction (qRT-PCR) assay, using iTAq Universal SYBR Green (Bio-Rad), according to the manufacturer’s instructions. Primers used for human cell lines were HsHamp forward, 5′-CCA-GCTGGA-TGC-CCA-TGT-T-3′, and reverse, 5′-GCC-GCA-GCA-GAA-AATGCA-3′; HsHprt1 forward, 5′-TGC-TTT-CCT-TGG-TCA-GGC-AG-3′, and reverse, 5′-AAG-CTT-GCG-ACC-TTG-ACC-AT-3′. The same procedure was used for mouse liver and the primers for quantitative real-time RT-PCR assay were: MmHamp1 forward, 5′-AAG-CAG-GGC-AGA-CAT-TGC-GAT-3′, and reverse, 5′-CAG-GAT-GTG-GCT-CTA-GGC-TAT-GT-3′; MmHprt1 forward, 5′-CTG-GTT-AAG-CAG-TAC-AGC-CCC-AA-3′, and reverse, 5′-CAGGAG-GTC-CTT-TTC-ACC-AGC-3′; MmId1 forward, 5′-ACC-CTG-AACGGC-GAG-ATC-A-3′, and reverse, 5′-TCG-TCG-GCT-GGA-ACA-CAT-G-3′.

### Statistical Analysis

Data are presented as mean ± standard error of mean (SD). Data of *in vitro* experiments are expressed as percentage or fold increase with respect to non-stimulated/non-treated cells. The data of *in vivo* experiments are expressed as percentage with respect to untreated animals and represented with box plots (for a better visualization of mice distribution in each group). Comparison of values between untreated and treated cells or mice was performed by two-tailed Student *t*-test for unpaired data. Differences were defined as significant for *P* < 0.05 or 0.001.

## Results

We previously found that unfractionated, glycol-split and oversulfated heparins are efficient suppressors of hepcidin. We also observed that high *N*-acetylation reduced the anti-hepcidin potency of these compounds, whereas oversulfation increased it (Poli et al., 2014a). However, other heparin properties for optimal anti-hepcidin activity remained to be characterized, in particular the molecular weight and the sulfation at the crucial 2-O and 6-O positions.

### Heparin Molecular Weight

**FIGURE 1:**
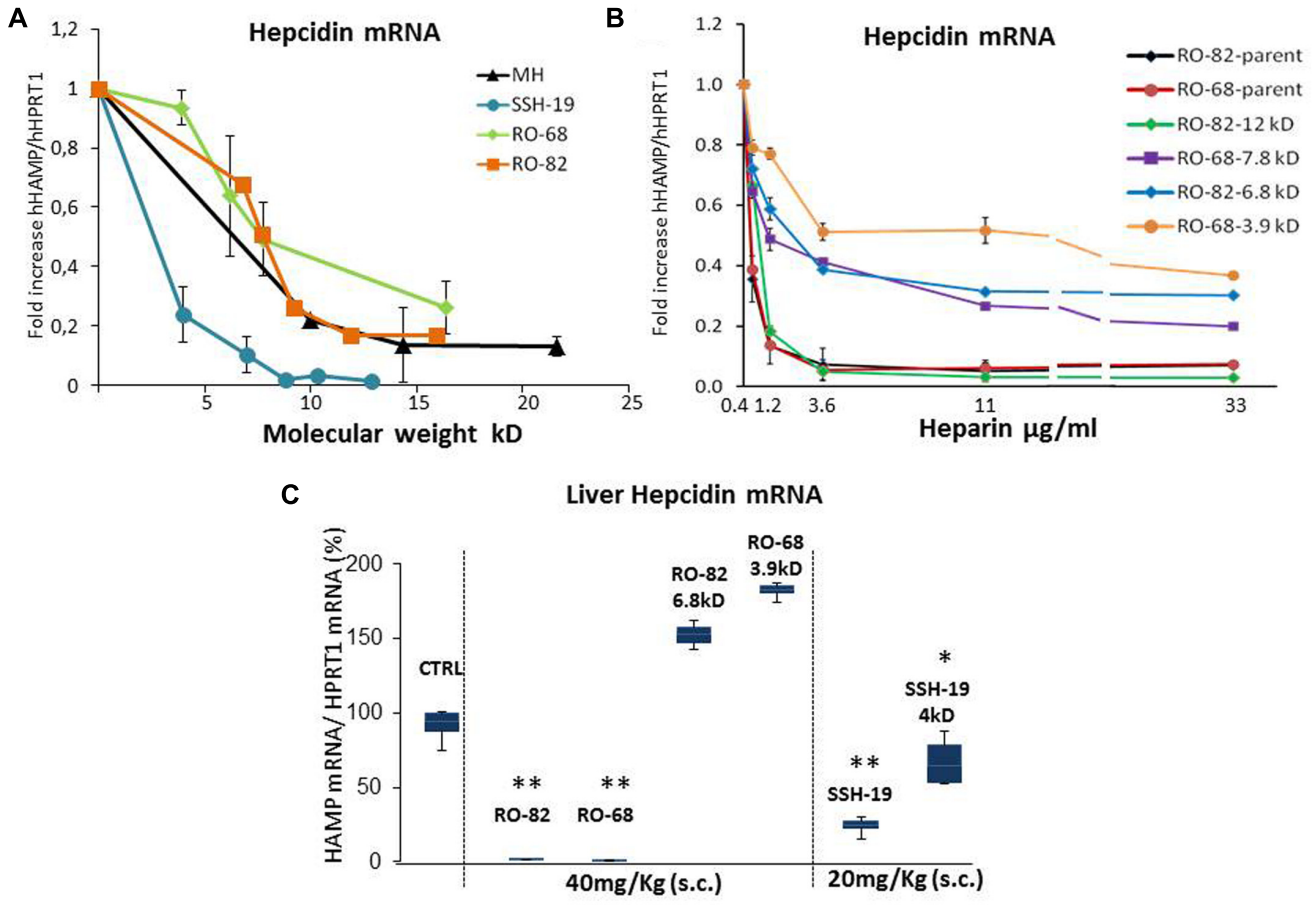
**Analysis of different heparin type fractions with different molecular weight. (A)** The fractions of the four different heparins, listed in **Table 1** were added to HepG2 cells at the concentration of 3.6 μg/ml for 6 h in presence of 10 ng/mL BMP6 and then the level of hepcidin mRNAs evaluated by qRT-PCR. The residual levels of hepcidin mRNA are plotted vs. the molecular weight of the fractions. **(B)** Dose-response curves of the two unfractionated RO heparins (RO-82 and RO-68), of their lightest fractions of 6.8 and 3.9 kD and of their highest fractions of 12 and 7.8 kD. HepG2 cells were treated with 0.4-1.2-3.6-11-33 μg/ml of different heparins for 6 h, in the presence of 10 ng/ml BMP6. The values were normalized for Hprt1 and the means were reported as fold increase of BMP6 stimulated cells, considered as control (value 1 in **A,B**). Three independent experiments for **(A,B)** graphs were performed and the induction of hepcidin expression in presence of BMP6 was reported as fold increase vs. untreated but stimulated cells. **(C)** The indicated compounds were injected subcutaneously in the mice at the concentrations of 40 mg/Kg for RO-82, RO-68; RO-82 6.8 kD, RO-68 3.9 kD and of 20 mg/ml for SSH-19 (SSLMWH-19) and SSH-19 4 kD. The mice were sacrificed after 6 h and the liver hepcidin mRNA quantified by qRT-PCR. The values were normalized for Hprt1 and reported in a box plot as a percentage of saline treated mice (CTRL).^∗^ and ^∗∗^ is a *P*-value <0.005 or 0.001 respectively.

To analyze the role of molecular weight, previously characterized heparins with high anti-hepcidin activity were fractionated by gel permeation chromatography. They include the unfractionated porcine mucosal heparin (MH), the glycol-split RO-82, the partially desulfated glycol-split RO-68 (Poli et al., 2014a) and the oversulfated SSLMWH-19 (Poli et al., 2014b). The chemical characteristics of the fractions are listed in **Table 1**. We collected and analyzed the lighter fractions of RO heparins because we wanted to define the minimum molecular weight with high anti-hepcidin activity, while for the oversulfated heparins that have a low mean molecular weight, we collected also the heavier fractions. To test their anti-hepcidin activity 3.6 μg/ml of the samples were added to HepG2 cells together with 10 ng/ml of BMP6, and after 6 h the hepcidin mRNA level quantified by qPCR. The residual level of hepcidin mRNA of the treated cells was plotted vs the molecular weight of each heparin fraction (**Figure 1A**). The potency of the four heparins decreased with the molecular weight. The plots of MH, RO-82 and RO-68 were similar and almost overlapping, with a strong decrease of potency below 7–10 kD. The oversulfated SSLMWH-19 was more potent, as reported before (Poli et al., 2014b) and it was inhibitory even at lower molecular weight, with a threshold of about 4 kD. The fractions of glycol-split heparins with lower molecular weight (RO-82 6.8 kD and RO-68 3.9 kD) could increase their activity at higher concentrations, thus we performed dose-response studies of these fractions in presence of BMP6 (10 ng/ml) for 6 h. The inhibition of hepcidin expression reached a plateau at 3.6 μg/ml for all heparins, but it accounted to about 50% for the low molecular weight fractions and for RO-68 7.8 kD versus a 90% of the parent preparations and the RO-82 12 kD (**Figure 1B**).

Next, we tested the heparin fractions *in vivo* by injecting them SC in mice and analyzing liver hepcidin mRNA after 6 h. Under these conditions the parental RO-82 and RO-68 at the dose of 40 mg/kg were fully inhibitory, as reported before (Poli et al., 2014a), while at the same dose their low molecular weight fractions of 3.9 and 6.8 kD showed no inhibition (**Figure 1C**). *In vivo* the RO-68 7.8 kD showed a similar anti-hepcidin activity to the RO-82 6.8 kD, whereas the RO-82 12 kD was as potent as the parent preparations (not shown). Interestingly, the oversulfated heparin (SSLMW-19) of 8.8 kD retained a strong activity even at the lower dose of 20 mg/kg causing about 90% suppression of hepcidin and also the lighter fraction of 4 kD retained the capacity to inhibit hepcidin (with a reduction of 50%). These results are in agreement with our previous report of a higher potency of the oversulfated heparins (Poli et al., 2014b).

### Sulfation at 2-O and 6-O

**FIGURE 2:**
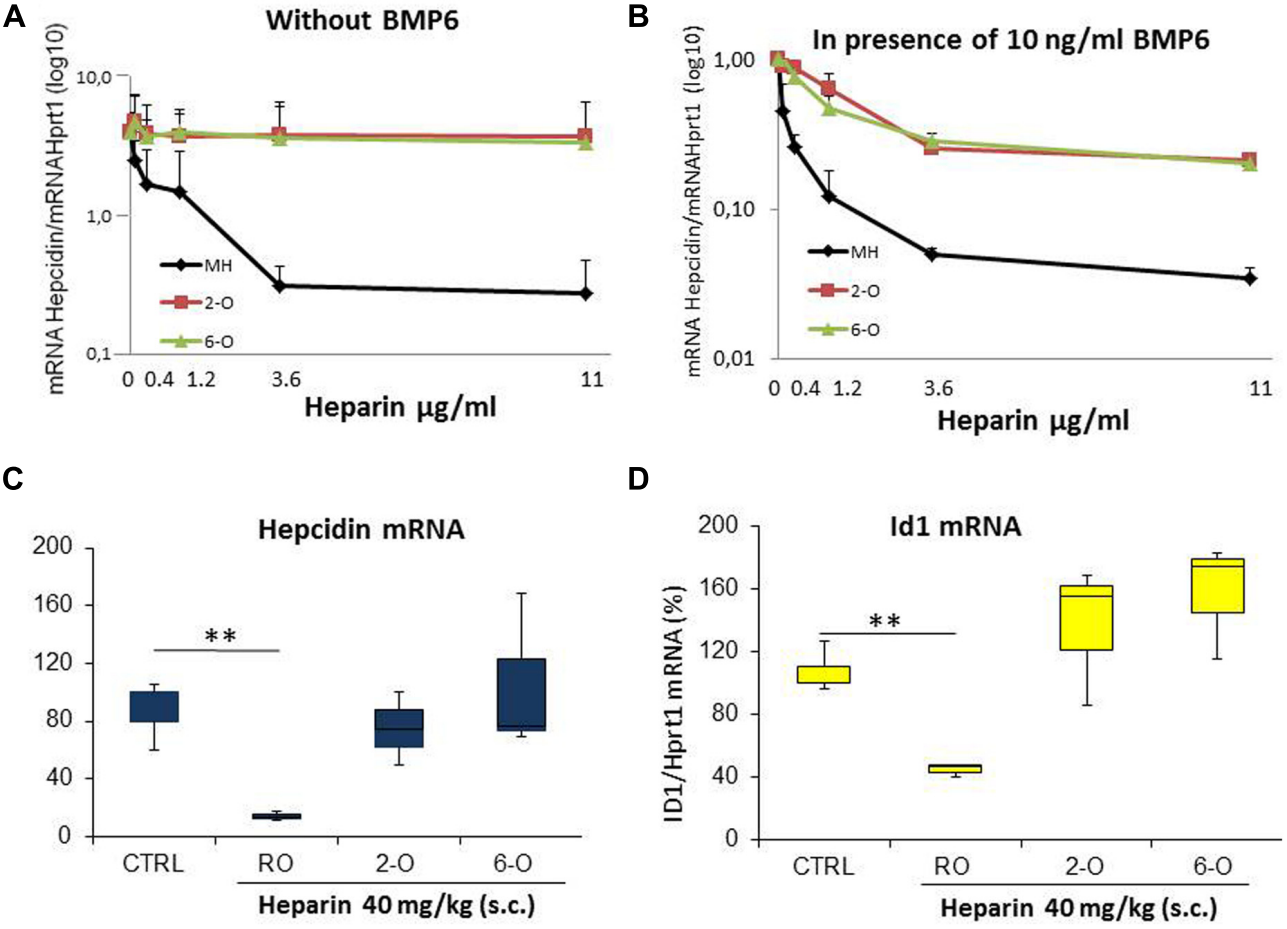
**Analysis of 2-O and 6-O desulfated mucosal heparins.** The unfractionated and unmodified Mucosal Heparin (MH) and of the two desulfated preparations (2-O and 6-O) were added to HepG2 at different concentrations (0.12-0.4-1.2-3.6-11 μg/ml) for 6 h without **(A)** or in presence **(B)** of BMP6 (10 ng/ml). The level of hepcidin mRNA was evaluated by qRT-PCR after 6 h of treatment and normalized for Hprt1. Three independent experiments for **(A,B)** were performed and the mean values expressed in logarithmic scale vs. untreated cells (value 1) were reported in the graphs. **(C,D)** The heparins (RO-82, 2-O and 6-O) were injected subcutaneously in the mice at the concentration of 40 mg/Kg. The mice were sacrificed after 6 h and the liver hepcidin and Id1 mRNA quantified by qRT-PCR. The values were normalized for Hprt1 and reported in a box plot as a percentage of saline treated mice (CRTL). Id1 was a control of the activation of the BMP/SMAD pathway. ^∗∗^ is a *P*-value <0.001.

Heparin binding activity is related not only to the density of sulfated groups but also on their spatial distribution, in particular their presence on the 2-O or the 6-O. Thus we tested the potency of inhibiting hepcidin expression in HepG2 of the heparins treated to remove sulfates from 2-O and from 6-O. The dose-response plots in **Figures 2A,B** showed that the hepcidin inhibitory activity of the two modified heparins is comparable and much lower of that of the control heparin (MH), both when the experiments were performed in the absence (**Figure 2A**) or the presence of 10 ng/ml BMP6 (**Figure 2B**). Then, we used these heparins at a dose of 40 mg/kg to treat the mice. The unfractionated RO heparin fully suppressed liver hepcidin mRNA after 6 h of treatment, while the 2-O and 6-O desulfated heparins showed no inhibitory activity at the same conditions (**Figure 2C**). The analysis of Id1 mRNA, considered a reliable index of activation of the BMP/SMAD pathway, confirmed the lack of activity of the two desulfated heparins (**Figure 2D**).

## Discussion

**FIGURE 3:**
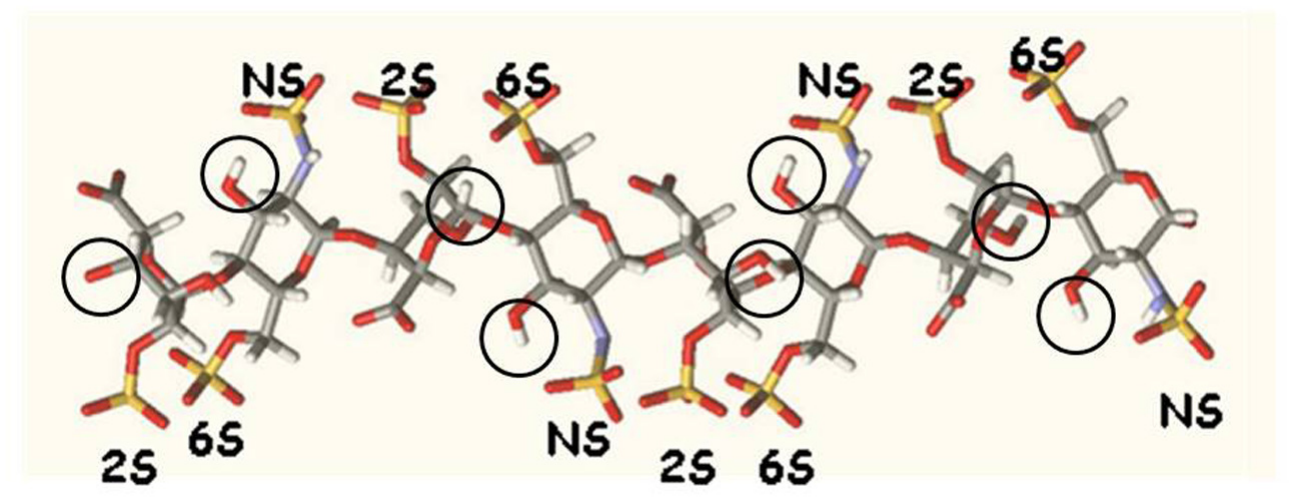
**Molecular model of an octasaccharide segment of heparin (Mulloy et al., 1993).** The structure shows a regular distribution of sulfated groups on both sides of the molecule. Circles indicate the position of the hydroxyl in position 3.

Heparins are promising anti-hepcidin agents that may find an application in the treatment of iron deficiencies linked to excess of hepcidin (Poli et al., 2014c). They are active also after the abolition/reduction of the anticoagulant property, typical of these compounds, facilitating their use *in vivo* and in chronic treatments. For this reason, an optimization of the molecular structure that inhibits hepcidin expression in the cells and in mice is important to improve the potency of this drug and also to obtain information on the mechanism of hepcidin inhibition. We have previously shown also that the oversulfation improves anti-hepcidin activity (Poli et al., 2014b). Moreover we have shown that the increase of *N*-acetylation decreases heparin activity, in line with the observation that *N*-acetylation reduces the degree of sulfation of the molecule (Poli et al., 2014a). In this work we explored the effect of molecular weight and of 2-O and 6-O sulfation on the anti-hepcidin activity, properties that were not analyzed in detail before. We separated by gel filtration various fractions of 4 different heparin types (one mucosal unmodified, two glycol split and one oversulfated heparins) with molecular weights ranging from about 20 kD to about 4 kD. We found that the anti-hepcidin activity of the unmodified or glycol-split heparins strongly decreased below about 10 kD, which corresponds to a 25-saccharide, while that of the oversulfated heparin dropped at molecular weight below 7 kD, which corresponds to ∼17-saccharide. This suggests that for maximal activity, the heparin should expose various highly sulfated binding sites, possibly acting by binding different molecules or different sites of the same molecules. In fact oversulfated heparins, even with molecular weight as low as 4 kD (∼10 saccharide) are able to reduce hepcidin *in vitro* and *in vivo*. The other major observation of this work is that the removal of the sulfates from the 2-O and 6-O positions have a negative dramatic effect on heparin activity. This indicates that both the residues are essential for the interaction and may suggest that the sequences needed for the interaction request a cluster of sulfate groups on the same size of the chain, and this, in the oversulfated can be reinforced by the presence of sulfate also in position 3 of glucosamine (see **Figure 3**). We also found an interesting parallelism between the heparin capacity to inhibit hepcidin mRNA expression in hepatic cells, and that to inhibit hepcidin mRNA when injected SC in the mice. Further studies are in progress to verify whether the oversulfated heparins act in alternative way by interacting with BMP receptors, as it was shown to occur in C2C12 and PC12 cells (Kuo et al., 2010).

## Conclusion

For optimal anti-hepcidin activity, both *in vitro* and *in vivo*, the heparins must have a high level of sulfation, which includes the sulfation of 2-O, 6-O and also *N*-Acetylation degree of about 14–14.6%, and a molecular weight above 6–10 kD, depending upon the level of sulfation.

## Author Contributions

MA participated in the execution of the experiments *in vitro* and *in vivo* and in the analysis of the results. She contributed also in the preparation of the paper. AN planned and coordinated the production and characterization of the compounds. She contributed in the preparation of the paper and she provided an important scientific support in the field of heparin structure. EE prepared and characterized the compounds. PR participated in the *in vitro* experiments using the compounds with different levels of sulfation. MD participated in the *in vitro* experiments using the compounds with different molecular weight. MG contributed in the processing and analysis of the samples derived from the *in vivo* experiments and in the preparation of the paper. PA contributed in the preparation of the paper and provided an important scientific support in the field of iron and hepcidin regulation. MP planned the *in vitro* and *in vivo* experiments, coordinated the project and contributed in the preparation of the paper.

## Conflict of Interest Statement

The authors declare that the research was conducted in the absence of any commercial or financial relationships that could be construed as a potential conflict of interest.

## Abbreviations

BMP: bone morphogenetic protein
SMAD: small mother against decapentaplegic
STAT: signal transducer and activator of transcription
IL-6: interleukin -6
